# Seasonal Changes in the Microbial Communities on Lettuce (*Lactuca sativa* L.) in Chungcheong-do, South Korea

**DOI:** 10.4014/jmb.2210.10001

**Published:** 2022-12-09

**Authors:** Woojung Lee, Min-Hee Kim, Juyeon Park, You Jin Kim, Eiseul Kim, Eun Jeong Heo, Seung Hwan Kim, Gyungcheon Kim, Hakdong Shin, Soon Han Kim, Hae-Yeong Kim

**Affiliations:** 1 Division of Food Microbiology, National Institute of Food and Drug Safety Evaluation, Ministry of Food and Drug Safety, Cheongju 28159, Republic of Korea; 2 Institute of Life Sciences and Resources and Department of Food Science and Biotechnology, Kyung Hee University, Yongin 17104, Republic of Korea; 3 Department of Food Science and Biotechnology, and Carbohydrate Bioproduct Research Center, College of Life Science, Sejong University, Seoul 05006, Republic of Korea

**Keywords:** 16S rRNA gene, lettuce, microbial community, metagenomics, potential pathogen

## Abstract

Lettuce is one of the most consumed vegetables worldwide. However, it has potential risks associated with pathogenic bacterial contamination because it is usually consumed raw. In this study, we investigated the changes in the bacterial community on lettuce (*Lactuca sativa* L.) in Chungcheong-do, South Korea, and the prevalence of foodborne pathogens on lettuce in different seasons using 16S rRNA gene-based sequencing. Our data revealed that the Shannon diversity index showed the same tendency in term of the number of OTUs, with the index being greatest for summer samples in comparison to other seasons. Moreover, the microbial communities were significantly different between the four seasons. The relative abundance of Actinobacteriota varied according to the season. Family *Micrococcaceae* was most dominant in all samples except summer, and *Rhizobiaceae* was predominant in the microbiome of the summer sample. At the genus level, the relative abundance of *Bacillus* was greatest in spring samples, whereas *Pseudomonas* was greatest in winter samples. Potential pathogens, such as *Staphylococcus* and *Clostridium*, were detected with low relative abundance in all lettuce samples. We also performed metagenome shotgun sequencing analysis on the selected summer and winter samples, which were expected to be contaminated with foodborne pathogens, to support 16S rRNA gene-based sequencing dataset. Moreover, we could detect seasonal biomarkers and microbial association networks of microbiota on lettuce samples. Our results suggest that seasonal characteristics of lettuce microbial communities, which include diverse potential pathogens, can be used as basic data for food safety management to predict and prevent future outbreaks.

## Introduction

Lettuce (*Lactuca sativa* L.) is one of the most widely consumed vegetables worldwide, including in South Korea, where from 2015 to 2020, lettuce production decreased in Gyeongsang-do and Gangwon-do, whereas it increased in Chungcheong-do [1]; In 2020, Chungcheong-do had the highest lettuce production (28,339 tons) in South Korea, followed by Jeolla-do (26,735 tons), and Gyeonggi-do (26,112 tons) [1]. Lettuce is low in calories, fat, and sodium and is a good source of fiber, iron, folate, and vitamin C. Moreover, lettuce exhibits anti-inflammatory, cholesterol-lowering, and anti-diabetic activities attributed to its bioactive compounds [2].

Recently, more foodborne disease outbreaks have been caused by fresh produce, including lettuce. Raw lettuce is consumed in salads, sandwiches, and hamburgers. In 2021, four foodborne disease outbreaks occurred through the packaged salad, and *Escherichia coli* O157:H7, *Listeria monocytogenes*, and *Salmonella enterica* subsp. *enterica* serovar Typhimurium were the causative foodborne pathogens [3]. Moreover, the Centers for Disease Control and Prevention (CDC) reported that in 2018 and 2019, foodborne disease outbreaks were caused by *E. coli* O157:H7 after the consumption of romaine lettuce in the USA [4]. Several studies have also reported that lettuce is contaminated by Shiga toxin-producing *E. coli* (STEC), *Bacillus cereus*, *L. monocytogenes*, *S. enterica* subsp. *enterica* serovar Typhimurium, *Staphylococcus aureus*, and *Campylobacter jejuni* [5-8].

Culture-dependent methods are typically used for pathogen detection. However, the use of culture-dependent methods for detecting various bacteria that can cause foodborne illnesses is limited because these methods are time-consuming and error-prone. Hence, culture-independent methods are used to directly analyze microbial communities from a sample [9-11]. Moreover, a culture-independent method can readily identify a large proportion of bacterial communities in a sample that are difficult to observe using culture-based methods. In previous study had also reported that high-throughput sequencing allows the identification of low-abundance bacteria in leafy vegetables that are not detected by culture-dependent methods [12]. Bacterial communities associated with fresh produce, such as sprouts, fruits, and vegetables, have also been analyzed using 16S rRNA gene sequencing [13-15]. Although several studies have investigated human pathogens in leafy vegetables [16-20], information on potential pathogens in lettuce with seasonal changes is limited. Thus, seasonal changes in the bacterial community composition of lettuce and the prevalence of foodborne pathogens in lettuce should be investigated using 16S rRNA gene-based sequencing analysis.

In this study, high-throughput sequencing was used to analyze seasonal changes in the microbial community composition of lettuce. In addition, seasonal variations in the lettuce microbiota were investigated to identify potential risks based on the presence of foodborne pathogens.

## Materials and Methods

### Collection and Preparation of Samples

A total of 86 lettuce samples (100 g/bundle) were harvested from Chungju, Cheongju, Nonsan, and Sejong in South Korea (two sites in Chungju, one site in Cheongju, two sites in Nonsan, and five sites in Sejong); they were collected in winter (February, 23 samples), spring (April, 23 samples), summer (August, 22 samples), and fall (October, 18 samples) in 2021. The sample collection sites (two sites in Chungju, one site in Cheongju, two sites in Nonsan, and five sites in Sejong) were chosen as locations where samples could be collected continuously for four seasons in order to minimize changes in the microbial community according to external environmental conditions such as agricultural water and compost. The samples were placed (100 g) in sterilized bags (FILTRA-BAG; Labplas, Canada) and mixed with 500 ml of buffered peptone water (Oxoid, UK). Using a BagMixer 400 (Interscience, Saint-Nom-la-Bretèche, France), the mixtures were homogenized for 10 min at 4 storke/s to detach the bacterial cells from the plant. The number of total bacteria was measured after incubating samples at 37°C for 24 h in 3M Petrifilm Aerobic Count Plates (3M Petrifilm, USA) with 1 ml of diluted bacterial cells. After incubation, cells were counted, and the following results were expressed as log CFU/g. The viable cell counts were carried out in triplicates.

### Bacterial Isolation

The detection of *B. cereus* and *S. aureus* was performed by modifying the Korea food code (No.2022-76, 2022.10.25). To isolate *B. cereus*, detected shotgun metagenomics sequencing samples were streaked on Mannitol egg yolk polymyxin agar plates (MYP, Oxoid, UK) and the plates were incubated for 24 h at 30°C. In addition, the samples were incubated with Tryptic soy broth (TSB, Oxoid, UK) containing 10% sodium chloride for 24 h at 37°C to isolate *S. aureus*. And then, incubated samples were streaked on Baird Parker agar plates (BPA, Oxoid) and the plates were incubated for 24 h at 37°C. Typical colonies of each bacteria were selected and purified to obtain single colonies. These colonies were identified using MALDI-TOF MS (BioMérieux, VITEK MS versus Bruker MALDI Biotyper, France).

### Extraction of Bacterial DNA

The genomic DNA of the lettuce samples was extracted as previously described [21]. The total genomic DNA from the lettuce samples was extracted using the DNeasy PowerSoil Kit (Qiagen, USA), following the manufacturer’s instructions. And the extracted DNA was purified using a DNeasy PowerClean Pro Cleanup Kit (Qiagen). DNA quality was assessed using a Qubit dsDNA HS Assay Kit (Thermo Fisher Scientific, Inc., USA), according to the manufacturer’s instructions.

### Bacterial 16S rRNA Gene-Based Sequencing and Metagenome Shotgun Sequencing

The extracted total DNA was amplified using primers 341F-TCGTCGGCAGCGTCAGATGTGTATAAG AGACAGCCTACGGGNGGCWGCAG and 805R-GTCTCGTGGGCTCGGAGATGTGTATAAGAGACAGGA CTACHVGGGTATCTAA TCC, which amplify the V3-V4 region of the 16S rRNA gene. PCR amplification was performed according to the protocol for preparing a 16S rRNA gene-based sequencing library using the MiSeq system (Illumina, Inc., USA) described in previous studies [16-20]. The library was quantified using a Qubit dsDNA HS Assay Kit (Thermo Fisher Scientific, Inc.). Library size and quality were evaluated using Bioanalyzer 2100 (Agilent, Inc.). Equimolar concentrations of each library from the different samples were pooled and sequenced using an Illumina MiSeq system (300 bp-paired ends), according to the manufacturer’s instructions.

Whole metagenome shotgun libraries were prepared using the Nextera DNA Flex Library Prep kit (Illumina) following the manufacturer’s instructions. The library was quantified using a Qubit 4.0 Fluorometer (Thermo Fisher Scientific, Inc.). The library size and quality were confirmed on a Bioanalyzer 2100 (Agilent, Inc.). Libraries were pooled and sequenced using the MiSeq system (300 bp-paired ends).

### Data Analysis

For microbiota community analysis, the nucleotide sequences of the V3-V4 region of the 16S rRNA gene were analyzed using the QIIME 2 pipeline (v2020.06) [22]. Raw sequence reads were quality-filtered, trimmed, and denoised using DADA2 [23]. The taxonomic position was assigned amplicon sequence variants (ASVs) using the classify-sklearn naïve Bayesian classifier against SILVA (v138) as the reference database. Qualified reads were reduced to the same number of reads per sample for normalization. Alpha diversity metrics (observed features and Shannon entropy) were estimated using q2-diversity. Beta diversity analysis was performed using the Vegan (v2.6-2) package [24] in R (v4.1.3). Principal coordinate analysis (PCoA) based on Bray-Curtis dissimilarity was conducted to compare microbiota composition among the samples. Linear discriminant analysis (LDA) effect size (LEFSe) analysis was performed to detect significant differences in bacterial classification (LDA score > 3.0)[25]. Data were visualized using the ggplot2 (v3.3.6) package [26] in R (v4.1.3). We constructed a correlated co-occurrence network using the Cytoscape plug-in conet (1.1.1 beta) based on the ensemble approach by combining the measures of correlations (Pearson, Spearman), mutual information similarities, and distance (Bray-Curtis and Kullback-Leibler). Co-occurrence patterns were visualized as networks using Cytoscape (3.9.1 v) with an implemented organic layout. The corresponding statistical analysis was performed using the implemented tool network analyzer.

The raw reads of whole metagenome shotgun sequencing were uploaded into CLC Genomics Workbench v22 (Qiagen), using the following options: Illumina import, paired-reads, and default distance options. The trimming was performed, and the de novo assembly tool was used for the assembly. Microbial genomics module was used for the microbial identification based on taxonomic profiling and find best matches using K-mer spectra.

### Statistical Analysis

Significant differences between the samples were analyzed using the Mann-Whitney U test, and the differences among samples were analyzed using the Kruskal-Wallis test in R (v4.1.3). Statistical significance was set at a *p*-value < 0.05. Differences in beta diversity were visualized using Principal Coordinates Analysis (PCoA) plots and tested by analysis of similarities (ANOSIM) based on the Bray-Curtis distance.

## Results and Discussion

### Abundance and Diversity of Bacterial Communities

A total of 5,752,269 reads (paired-end, Phred ≥ Q20) were obtained from the lettuce samples (*n* = 86). The average read number per sample was 16,580 ± 8,963, and these reads were binned into 2,432 types of features. Usually, a high abundance (> 90%) of Cyanobacteria at the phylum level, present in the raw sequencing results, indicates the occurrence of contaminating sequences from a plant mitochondrial and chloroplast DNA. In our results, Cyanobacteria was the predominant bacterial phylum in all lettuce samples (*i.e.*, in all samples harvested in all seasons). Moreover, all lettuce samples were predominantly composed of chloroplast (88.85%, 81.77%, 90.29%, and 89.24%) and mitochondrial (2.06%, 1.90%, 3.86%, and 4.30%) DNA (Fig. S1). To exclude the interference of contaminating sequences, sequence reads from Cyanobacteria at the phylum level, including the sequences pertaining to plant chloroplasts and mitochondria, were removed from the data before analyzing microbial diversity. Although there is a loss of read numbers when deleting chloroplast-related sequences, it was confirmed that the 16S rRNA sequencing-based bacterial structure of the chloroplast-removed samples was more comparable to the metagenome-based bacterial structure, in relation to unfiltered 16S rRNA sequencing dataset (Fig. S2).

**Fig. 1:**
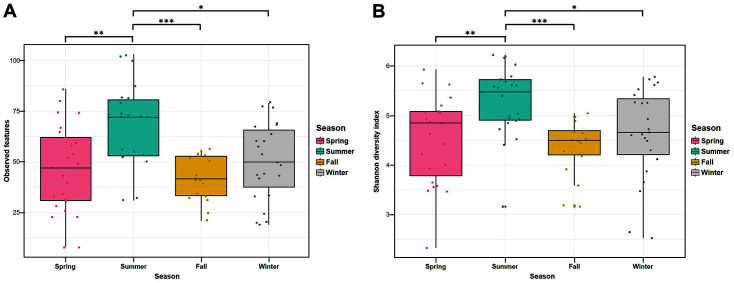
Comparison of observed features and Shannon diversity in the lettuce samples collected in different seasons. Significance of the difference was determined using Mann-Whitney U-test (*p < 0.05, **p < 0.01, ***p < 0.001).

As shown in Fig. 1, the average number of observed features in the summer samples (67.50 ± 20.49) was higher than that in the spring (47.17 ± 20.84, *p* < 0.0043), fall (41.94 ± 10.91, *p* < 0.0001), and winter samples (50.13 ± 19.01, *p* < 0.0102). The Shannon diversity indices of the summer samples (5.27 ± 0.72) were significantly higher than those of the spring (4.52 ± 0.89, *p* < 0.0078), fall (4.37 ± 0.56, *p* < 0.0001), and winter samples (4.64 ± 0.92, *p* < 0.0133). In this study, the number of total bacteria was measured in 3M Petrifilm Aerobic Count Plates. As a result of comparing the number of total bacteria according to the four seasons, the summer samples were significantly different between the four seasons. (Fig. S3). The average number of total bacteria in summer lettuce samples was 5.6 ± 0.06 log CFU/g, which was higher than in other seasons. This result is consistent with 16S rRNA gene-based sequencing (Fig. 1) that the microbial diversity of summer samples was higher than in other seasons. However, no significant differences were observed between the sampling sites (Chungju, Cheongju, Nonsan, and Sejong), which were adjacent to each other, which is consistent with the results of previous studies showing bacterial diversity and population characteristics in Chinese cabbage [16], lettuce [17], and perilla [18]. Moreover, the average temperature of summer (26°C) was higher than that of spring, fall, and winter. These results suggest that the bacterial community in lettuce differs according to environmental factors, such as temperature and relative humidity.

### Comparison of Microbial Community among Lettuce Samples

The hypervariable regions of the 16S rRNA gene are used to detect microbial communities in a sample, typically down to the genus level. In the current study, for each lettuce sample, the amplicons were sequenced for 5,752,269 reads, of which 1,425,849 were mapped.

**Fig. 2:**
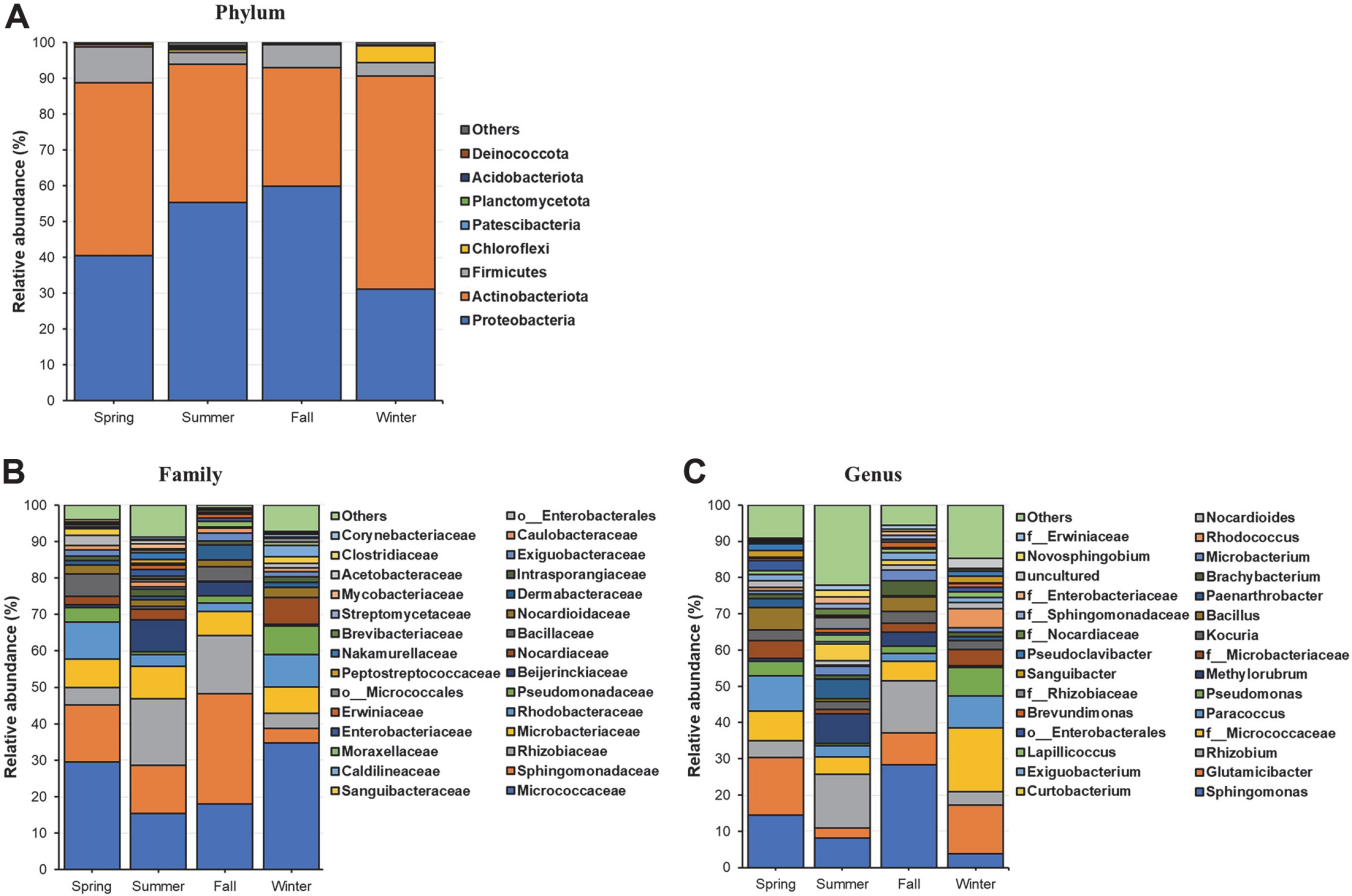
Relative abundance of various bacterial taxa at the A phylum, B family, and C genus levels in the lettuce samples collected during different seasons.

A total of 14 phyla, 35 classes, 146 families, and 226 genera were identified in all lettuce samples used in this study. The relative abundances of the microbiota (phylum, family, and genus levels) from lettuce samples harvested in each season are shown in Fig. 2.

Proteobacteria and Actinobacteriota were the predominant bacterial phyla in all lettuce samples. Proteobacteria exhibited the highest relative abundance in the fall samples (59.89%), followed by that in the summer (55.30%), spring (40.49%), and winter (31.11%) samples. The highest relative abundance of Actinobacteriota was observed in the winter samples (59.46%), followed by that in the spring (48.31%) and summer (38.57%) samples. The relative abundance of Firmicutes in the summer, spring, and winter samples was as follows: 3.30%, 9.99%, and 3.78%, respectively (Fig. 2A). According to previous studies, the phyla Proteobacteria, Actinobacteriota, and Firmicutes comprise the majority of bacteria on the leaf surface (phyllosphere) of other agricultural and native plant species [27].

Family level microbiota composition of the lettuce samples is presented in Fig. 2B. At the family level, the microbiota of the spring samples (more than 5% of the total sequences) was mainly composed of *Micrococcaceae* (29.54%), *Sphingomonadaceae* (15.58%), *Rhodobacteraceae* (10.11%), *Microbacteriaceae* (7.84%), and *Bacillaceae* (6.18%). The microbiota of the summer and fall samples was predominantly composed of *Rhizobiaceae* (18.30% and 15.94%, respectively), *Micrococcaceae* (15.46% and 17.94%, respectively), *Sphingomonadaceae* (13.07% and 30.36%, respectively), and *Microbacteriaceae* (8.99% and 6.62%, respectively). *Micrococcaceae* (34.70%), *Rhodobacteraceae* (8.85%), *Pseudomonadaceae* (7.86%), *Nocardiaceae* (7.24%), and *Microbacteriaceae* (7.24%) were the predominant families detected in the winter samples. These family groups are commonly found in a large variety of terrestrial and aquatic ecosystems, including soil, fresh and marine water, sand, and vegetation. Diverse potential pathogens, such as those belonging to *Clostridiaceae*, *Streptomycetaceae*, *Bacillaceae*, *Enterobacteriaceae*, and *Pseudomonadaceae*, were also detected in our study, but their relative abundance varied with the seasons. Comparative analysis of the Illumina sequencing data based on seasons revealed that the relative abundance of *Bacillaceae* was significantly higher in the spring samples (April) than in the samples from other seasons, whereas the relative abundance of *Enterobacteriaceae* was the highest in the summer samples (August). Furthermore, the relative abundance of *Pseudomonadaceae* was the highest in the winter (February) samples. According to previous studies, *Bacillaceae*, *Enterobacteriaceae*, and *Pseudomonadaceae* are the dominant families reported in lettuce [14, 17]. Moreover, previous studies on microbial communities in lettuce roots reported that *Pseudomonadaceae* was the core family, which correlated with the family *Sphingomonadaceae* [28].

At the genus level, the lettuce microbiota exhibited diverse composition in samples from different seasons. *Bacillus*, *Pseudomonas*, and *Sphingomonas* were found in all lettuce samples. *Sphingomonas* was the most remarkably enriched genus in the fall samples. The relative abundance of *Bacillus* was significantly higher in the spring samples than in those from other seasons, whereas the relative abundance of *Pseudomonas* was the highest in the winter (February) samples (Fig. 2C). Moreover, the bacterial genera *Rhizobium*, *Glutamicibacter*, *Paracoccus*, and *Methylorubrum* were commonly found in natural environments (Fig. 2C). Diverse potential pathogens, such as *Staphylococcus* and *Clostridium*, with low relative abundances, were also detected. *Staphylococcus* and *Clostridium* were present in the lettuce samples collected in spring, summer, and winter. In the fall samples, the presence of the genus *Clostridium* was confirmed (Fig. S4).

The potential human pathogenicity of *Bacillus* spp. should not be neglected because these bacteria are increasingly isolated from hospitalized patients, despite being one of the most prevalent microorganisms in nature [29]. The pathogenic potential of bacteria is associated with several virulence factors. Additionally, *Pseudomonas* is a potential human pathogen, which has been observed in large proportions in the bacterial communities of cold-stored leafy vegetables, such as spinach and chard, red cabbage, and mixed salads [14, 19]. The genus *Sphingomonas* has been detected in plants and soil and can act as a protective microbe for plants by suppressing disease symptoms and decreasing pathogen growth [30, 31]. It can also degrade organic pollutants and promote plant growth.

**Fig. 3:**
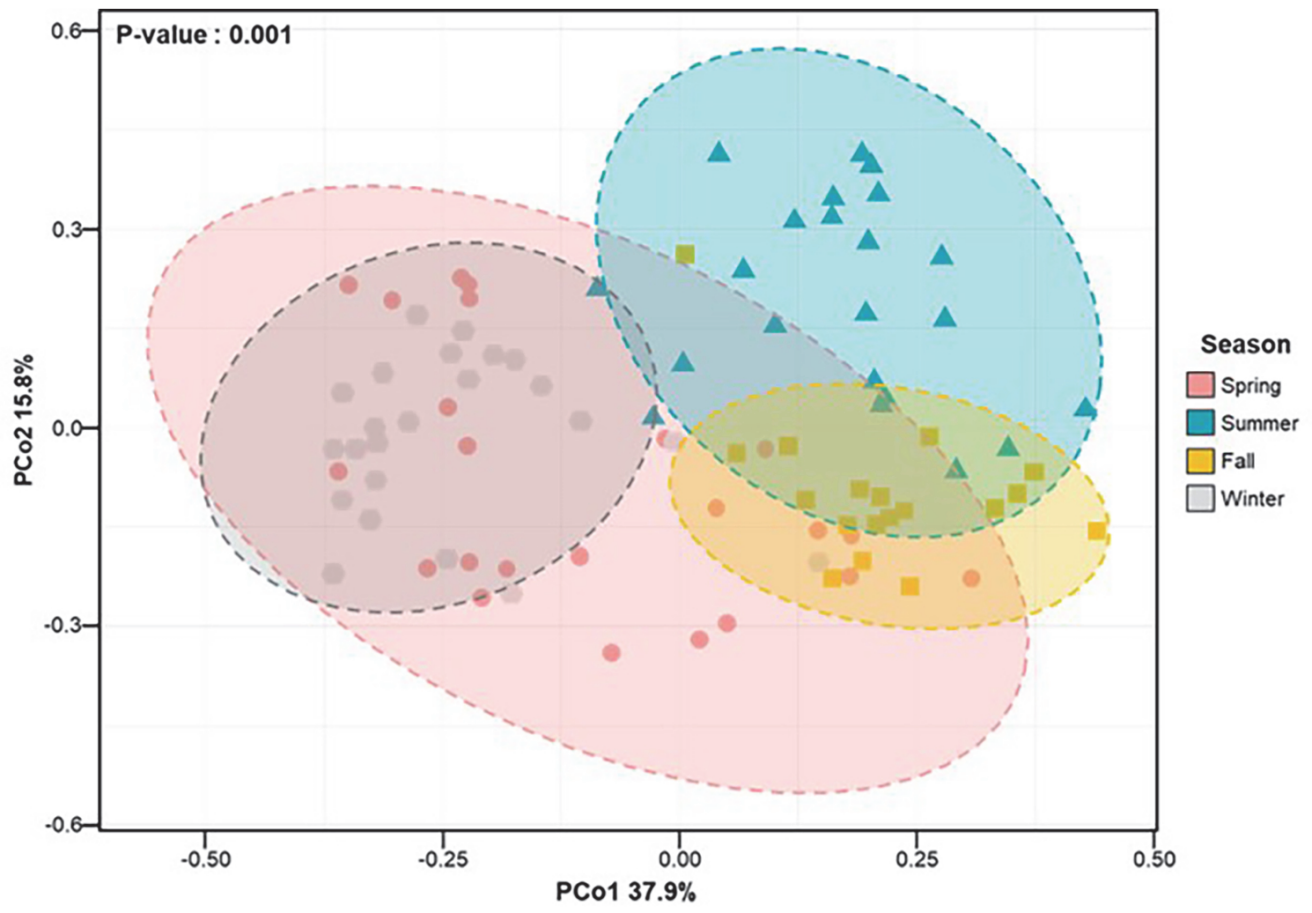
Principal coordinates analysis (PCoA) plot based on Bray-Curtis dissimilarity according to different seasons.

Beta diversity was illustrated using PCoA based on Bray-Curtis dissimilarity, which showed similarities in bacterial composition across samples from different seasons. The variation in bacterial composition based on the four seasons in the lettuce samples was statistically significant. The bacteria in the lettuce samples represented four clusters according to the sampling time (spring, summer, fall, winter) (ANOSIM, *p* = 0.001; Fig. 3). Bacterial structures in the lettuce samples were significantly separated into four seasonal groups (unweighted, weighted UniFrac distance, *p* < 0.05). The distance within the samples of the fall group was smaller than that within the samples from other seasons. The groups were significantly separated (*p* < 0.05), except for spring and summer samples and summer and winter samples. (Fig. S5). These results showed that the bacterial communities of lettuce samples have a close relationship with sampling time.

### Season-Specific Bacterial Taxa of Lettuce

**Fig. 4:**
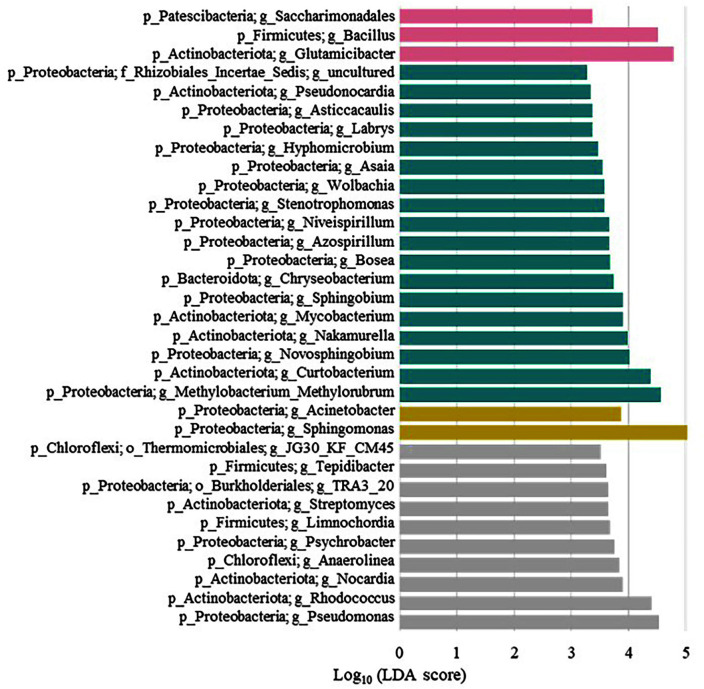
Linear discriminant analysis Effect Size (LEfSe) for the bacterial communities. The LEfSe plot shows enriched bacterial genera significantly associated with the four seasons. Three genera were enriched in the spring (pink), 18 in the summer (blue), two in the fall (yellow), and 10 in the winter (gray) samples. The linear discriminant analysis score is shown at the logarithmic scale underneath the bars.

To identify distinguishing taxa in the lettuce samples for all four seasons, the LEfSe method was used (Fig. 4). In our 16S rRNA gene datasets, 2–18 biomarker taxa were identified in the samples from different seasons. Significant major genera were detected, including *Bacillus* and *Glutamicibacter* in the spring samples; *Mycobacterium*, *Novosphingobium*, *Curtobacterium*, and *Methylorubrum* in the summer samples; *Acinetobacter* and *Sphingomonas* in the fall samples; and *Nocardia*, *Rhodococcus*, and *Pseudomonas* in the winter samples (LDA > 3.0).

**Fig. 5:**
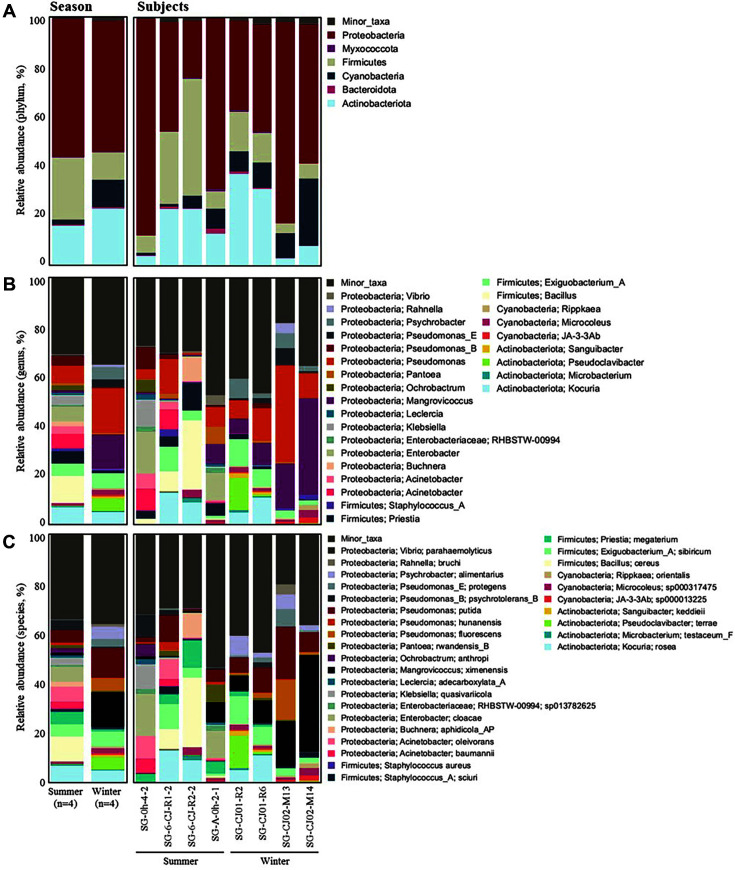
Relative abundance of various bacterial taxa at the A phylum, B genus, and C species levels in the lettuce samples collected during different seasons based on metagenome shotgun sequencing.

The summer (*n* = 4) and winter (*n* = 4) samples, which were anticipated to be contaminated with foodborne pathogens, were subjected to further metagenome analysis due to the pronounced differences in bacterial structure between the two seasons. (Table S1 and Fig. 5). Species-level identification analysis showed bacterial structures in the lettuce samples were significantly separated between the summer and winter samples, supporting the results based on 16S rRNA sequencing analysis. A metagenome-assembled genome (MAG) predicted to be *B. cereus* was identified in seven samples (except one summer sample) and *S. aureus* was identified in 4 samples (two samples per season). Also, we performed an experiment to isolate *B. cereus* and *S. aureus*, food-poisoning bacteria that can be most frequently contaminated in lettuce samples and the environments (soil and water). We could isolate *B. cereus* strains and *S. aureus* strains from related-MAG detected samples using the selective medium. In functional analysis, while *B. cereus*-related virulence genes (*entFM*, *bceT*, *groEL*, and *hblC*) were only detected in summer samples, *S. aureus*-related toxin genes were not found in summer and winter samples (data not shown).

Specifically, we identified nine potentially pathogenic genera associated with human health in lettuce samples harvested from different seasons. *Bacillus* was detected as a spring-specific taxon. Although the genera *Bacillus*, *Pseudomonas*, and *Sphingomonas* are generally considered nonpathogenic, several species have been reported as human pathogens.

In the summer samples, genera *Stenotrophomonas*, *Chryseobacterium*, *Mycobacterium*, and *Methylobacterium* were identified as specific biomarkers. The genus *Stenotrophomonas* is ubiquitous and is particularly closely associated with plants. These species play an important ecological role in the nitrogen and sulfur cycles [32]. Additionally, several *Stenotrophomonas* spp., particularly *S. maltophilia* and *S. rhizophila*, can engage in beneficial interactions with plants. However, *S. maltophilia* is an emerging pathogen responsible for fatal human infections [33]. The genus *Chryseobacterium* occurs widely in environmental, food, and water sources, and many species are resistant to several antimicrobials [34]. Several *Chryseobacterium* spp. have been implicated in human diseases and have caused disease outbreaks via contaminated water [35]. The genus *Mycobacterium* encompasses a large group of gram-positive, rod-shaped, acid-fast organisms belonging to the phylum Actinobacteria. *Mycobacterium* spp. inhabit a diverse range of environments, including water and soil [36]. In addition, many *Mycobacterium* spp. are well-known human pathogens, especially *Mycobacterium tuberculosis* and *Mycobacterium leprae*, which are human pathogens responsible for tuberculosis and leprosy, respectively [37]. The genus *Methylobacterium* is ubiquitously present in a wide variety of habitats, including air, soil, freshwater, and sediments, and can exist either in free form or in association with plant tissues [38]. However, *Methylobacterium* spp. are rarely found in human clinical samples as opportunistic pathogens [39].

We identified the *Acinetobacter* and *Sphingomonas* genera as fall-specific taxa. *Acinetobacter* occupies an important position in nature because of its ubiquitous presence in diverse environments, such as soils, water, sediments, and contaminated sites. In addition to the ecological importance of *Acinetobacter*, *Acinetobacter baumannii*, a pathogenic strain, causes a wide range of hospital-acquired infections, especially in intensive care units [40].

*Pseudomonas* and *Rhodococcus* were detected as winter-specific biomarkers. *Rhodococcus* spp. are soil-borne organisms that are widespread in the environment. They have been isolated from the feces of various animal species, including equines, bovines, small ruminants, dogs, pigs, and wildlife. Exposure to soil contaminated with herbivore manure is likely the major route of both animal and human infection. *Rhodococcus equi* is a facultative intracellular bacterial pathogen found in horses and other domestic animals, and it acts as an opportunistic pathogen in humans [41].

**Fig. 6:**
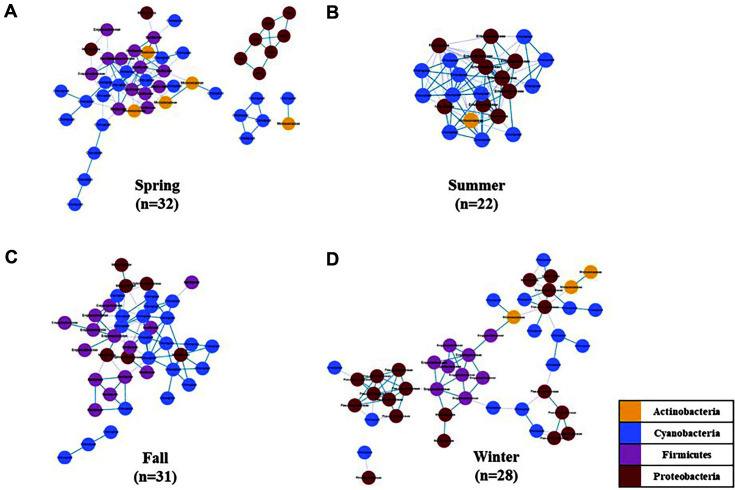
Co-occurrence network plots of bacterial taxa within the microbiota of lettuce by season. The cooccurrence networks revealed correlations of ASVs in lettuce samples obtained in **A** spring, **B** summer, **C** fall, and **D** winter. The network plot is expressed in different colors according to the phylum level and by a circle at the family level. Edges connecting the nodes in blue indicate a positive correlation (Co-presence), whereas the edges in gray indicate a negative correlation (Mutual-exclusion).

To identify specific biomarkers according to the four different seasons, a correlated co-occurrence network analysis was performed (Fig. 6). Four phyla, namely, Actinobacteria, Cyanobacteria, Firmicutes, and Proteobacteria, were involved in the network configuration, which exhibited different configurations for each season. In the summer samples, with 18 biomarker taxa, the network configuration involving only Actinobacteria, Cyanobacteria, and Proteobacteria was the simplest in relation to other seasons. In the fall samples, a network involving Actinobacteria, Cyanobacteria, and Firmicutes was formed, whereas, in the spring and winter samples, all four phyla were involved.

In this study, we confirmed seasonal changes in the microbial communities of lettuce in South Korea using metagenomics. The microbial community composition differed significantly among the four seasons. However, no significant differences were observed between the sampling sites (Chungju, Cheongju, Nonsan, and Sejong) that were adjacent to each other. In particular, the lettuce microbiome contained bacterial genera with known opportunistic human pathogenic species, such as *Bacillus*, *Pseudomonas*, and *Sphingomonas*, regardless of the sampling season. In addition, diverse potential pathogens, such as *Staphylococcus* and *Clostridium*, were detected with low relative abundance. Our results suggest that seasonal characteristics of lettuce microbial communities, which include diverse potential pathogens, can be used as base data for food safety management to predict and prevent future outbreaks. In future studies, the changes in microbial communities and diverse potential pathogens during household washing and refrigerated storage should be investigated for the safe intake of lettuce.

## Supplemental Materials

Supplementary data for this paper are available on-line only at http://jmb.or.kr.

## References

[1] Korean Statistical Information Service https://kosis.kr/statHtml/statHtml.do?orgId=101&tblId=DT_1ET0028&vw.

[2] Kim MJ, Moon Y, Tou JC, Mou B, Waterland NL (2016). Nutritional value, bioactive compounds and health benefits of lettuce (*Lactuca sativa* L.). J. Food Compos. Anal..

[3] Centers for Disease Control and Prevention (2021). List of selected multistate foodborne outbreak investigations.

[4] Hoff C, Higa J, Patel K, Gee E, Wellman A, Vidanes J (2021). An outbreak of *Escherichia coli* O157:H7 infections linked to romaine lettuce exposure - United States, 2019. MMWR Recomm. Reps..

[5] Marshall KE, Hexemer A, Seelman SL, Fatica MK, Blessington T, Hajmeer M (2020). Lessons learned from a decade of investigations of shiga toxin-producing *Escherichia coli* outbreaks linked to leafy greens, United States and Canada. Emerg. Infect. Dis..

[6] Kim YJ, Kim HS, Kim KY, Chon JW, Kim DH, Seo KH (2016). High occurrence rate and contamination level of *Bacillus cereus* in organic vegetables on sale in retail markets. Foodborne Pathog. Dis..

[7] Jackson KA, Stroika S, Katz LS, Beal J, Brandt E, Nadon C (2016). Use of whole genome sequencing and patient interviews to link a case of sporadic listeriosis to consumption of prepackaged lettuce. J. Food Prot..

[8] Heaton JC, Jones K (2008). Microbial contamination of fruit and vegetables and the behaviour of enteropathogens in the phyllosphere: a review. J. Appl. Microbiol..

[9] Hugenholtz P, Tyson GW (2008). Metagenomics. Nature.

[10] Ryu JA, Kim E, Yang SM, Lee S, Yoon SR, Jang KS (2021). High-throughput sequencing of the microbial community associated with the physicochemical properties of meju (dried fermented soybean) and doenjang (traditional Korean fermented soybean paste). LWY.

[11] Ryu JA, Kim E, Kim MJ, Lee S, Yoon SR, Ryu JG (2021). Physicochemical characteristics and microbial communities in gochujang, a traditional Korean fermented hot pepper paste. Front. Microbiol..

[12] Jackson CR, Randolph KC, Osborn SL, Tyler HL (2013). Culture dependent and independent analysis of bacterial communities associated with commercial salad leaf vegetables. BMC Microbiol..

[13] Asakura H, Tachibana M, Taguchi M, Hiroi T, Kurazono H, Makino SI (2016). Seasonal and growth-dependent dynamics of bacterial community in radish sprouts. J. Food Saf..

[14] Leff JW, Fierer N (2013). Bacterial communities associated with the surfaces of fresh fruits and vegetables. PLoS One.

[15] Kim E, Cho EJ, Yang SM, Kim MJ, Kim HY (2021). Novel approaches for the identification of microbial communities in kimchi: MALDI-TOF MS analysis and high-throughput sequencing. Food Microbiol..

[16] Kim D, Hong S, Kim YT, Ryu S, Kim HB, Lee JH (2018). Metagenomic approach to identifying foodborne pathogens on chinese cabbage. J. Microbiol. Biotechnol..

[17] Yu YC, Yum SJ, Jeon DY, Jeong HG (2018). Analysis of the microbiota on lettuce (*Lactuca sativa* L.) cultivated in South Korea to identify foodborne pathogens. J. Microbiol. Biotechnol..

[18] Jeon DY, Yum SJ, Seo DW, Kim SM, Jeong HG (2019). Leaf-associated microbiota on perilla (*Perilla frutescens* var. frutescens) cultivated in South Korea to detect the potential risk of food poisoning. Food Res. Int..

[19] Tatsika S, Karamanoli K, Karayanni H, Genitsaris S (2019). Metagenomic characterization of bacterial communities on ready-to-eat vegetables and effects of household washing on their diversity and composition. Pathogens.

[20] Seo DW, Yum SJ, Lee HR, Kim SM, Jeong HG (2022). Microbiota analysis and microbiological hazard assessment in chinese chive (*Allium tuberosum* Rottler) depending on retail types. J. Microbiol. Biotechnol..

[21] Gu G, Ottesen A, Bolten S, Ramachandran P, Reed E, Rideout S (2018). Shifts in spinach microbial communities after chlorine washing and storage at compliant and abusive temperatures. Food Microbiol..

[22] Bolyen E, Rideout JR, Dillon MR, Bokulich NA, Abnet CC, Al-Ghalith GA (2019). Reproducible, interactive, scalable and extensible microbiome data science using QIIME 2. Nat. Biotechnol..

[23] Callahan BJ, McMurdie PJ, Rosen MJ, Han AW, Johnson AJA, Holmes SP (2016). DADA2: High-resolution sample inference from Illumina amplicon data. Nat. Methods.

[24] Oksanen J, Simpson GL, Blanchet FG, Kindt R, Legendre P, Minchin PR (2022). Vegan: community ecology package R package, version 2.6-2.

[25] Segata N, Izard J, Waldron L, Gevers D, Miropolsky L, Garrett WS (2011). Metagenomic biomarker discovery and explanation. Genome Biol..

[26] Villanueva RAM, Chen ZJ (2019). ggplot2: Elegant graphics for data analysis (2nd ed). Meas. Interdiscip. Res. Perspect..

[27] Lopez-Velasco G, Carder PA, Welbaum GE, Ponder MA (2013). Diversity of the spinach (*Spinacia oleracea*) spermosphere and phyllosphere bacterial communities. FEMS Microbiol. Lett..

[28] Knief C, Delmotte N, Chaffron S, Stark M, Innerebner G, Wassmann R (2012). Metaproteogenomic analysis of microbial communities in the phyllosphere and rhizosphere of rice. ISME J..

[29] Celandroni F, Salvetti S, Gueye SA, Mazzantini D, Lupetti A, Senesi S (2016). Identification and pathogenic potential of clinical *Bacillus* and *Paenibacillus* isolates. PLoS One.

[30] Innerebner G, Knief C, Vorholt JA (2011). Protection of *Arabidopsis thaliana* against leaf-pathogenic *Pseudomonas syringae* by *Sphingomonas* strains in a controlled model system. Appl. Environ. Microbiol..

[31] Kim H, Nishiyama M, Kunito T, Senoo K, Kawahara K, Murakami K (1998). High population of *Sphingomonas* species on plant surface. J. Appl. Microbiol..

[32] Ryan RP, Monchy S, Cardinale M, Taghavi S, Crossman L, Avison MB (2009). The versatility and adaptation of bacteria from the genus *Stenotrophomonas*. Nat. Rev. Microbiol..

[33] Denton M, Kerr KG (1998). Microbiological and clinical aspects of infection associated with *Stenotrophomonas maltophilia*. Clin. Microbiol. Rev..

[34] Kirby JT, Sader HS, Walsh TR, Jones RN (2004). Antimicrobial susceptibility and epidemiology of a worldwide collection of *Chryseobacterium* spp. Report from the SENTRY antimicrobial surveillance program (1997-2001). J. Clin. Microbiol..

[35] Hoque SN, Graham J, Kaufmann ME, Tabaqchali S (2001). *Chryseobacterium* (*Flavobacterium*) *meningosepticum* outbreak associated with colonization of water taps in a neonatal intensive care unit. J. Hosp. Infect..

[36] Gao B, Gupta RS (2012). Phylogenetic framework and molecular signatures for the main clades of the phylum *Actinobacteria*. Microbiol. Mol. Biol. Rev..

[37] Medjahed H, Gaillard JL, Reyrat JM (2010). *Mycobacterium abscessus*: A new player in the mycobacterial field. Trends Microbiol..

[38] Bijlani S, Singh NK, Eedara VVR, Podile AR, Mason CE, Wang CCC (2021). *Methylobacterium ajmalii* sp. nov., isolated from the international space station. Front. Microbiol..

[39] Green PN, Ardley JK (2018). Review of the genus *Methylobacterium* and closely related organisms: A proposal that some *Methylobacterium* species be reclassified into a new genus, *Methylorubrum* gen. nov. Int. J. Syst. Evol. Microbiol..

[40] Fiester SE, Actis LA (2013). Stress responses in the opportunistic pathogen *Acinetobacter baumannii*. Future Microbiol..

[41] Vail KJ, da Silveira BP, Bell SL, Cohen ND, Bordin AI, Patrick KL (2021). The opportunistic intracellular bacterial pathogen *Rhodococcus equi* elicits type I interferon by engaging cytosolic DNA sensing in macrophages. PLoS Pathog..

