# Supplementary material for: Seasonal Changes in the Microbial Communities on Lettuce (Lactuca sativa L.) in Chungcheong-do, South Korea
Source: J Microbiol Biotechnol. 2022 Dec 9;33(2):219–27. doi: 10.4014/jmb.2210.10001 (PMC9998205; doi:10.4014/jmb.2210.10001)
Supplement: Supplementary file 1 [file jmb-33-2-219-supple.pdf]

J. Microbiol. Biotechnol.
https://doi.org/10.4014/jmb.2210.10001

Table S1. Summary of analyzed metagenome shotgun sequence information.

Sample	Season	No. of reads	Avg. length	No. of reads after trim	% Trimmed	Avg. length after trim	No. of features
0h-4-2_S1	Summer	10,829,594	246.3	10,811,704	99.83	234.9	210
6-CJ-R1-2_S2	Summer	9,182,086	260.7	9,175,594	99.93	242.1	315
6-CJ-R2-2_S3	Summer	8,971,656	255.1	8,963,927	99.91	241.1	262
A-0h-2-1_S4	Summer	7,964,866	245.7	7,954,126	99.87	232.6	295
CJ01-R2_S1	Winter	7,981,914	260.3	7,978,013	99.95	245	325
CJ01-R6_S2	Winter	9,582,472	254.7	9,576,491	99.94	243.8	328
CJ02-M13_S3	Winter	10,680,326	258.2	10,675,424	99.95	244.8	162
CJ02-M14_S4	Winter	9,517,026	257.7	9,511,638	99.94	247.3	188

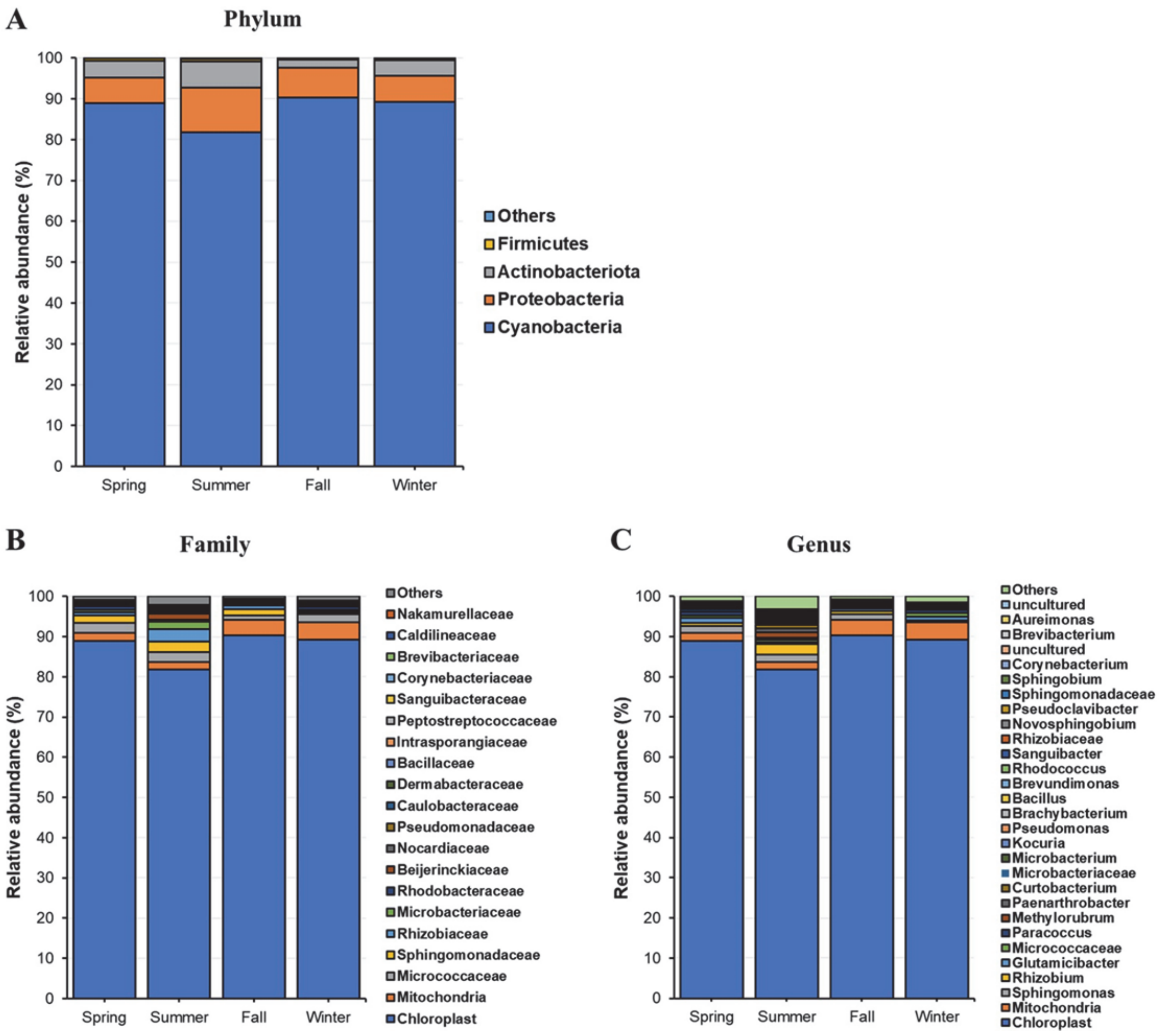

Fig. S1. Relative abundance of various bacterial taxa at the **A** phylum, **B** family, and **C** genus levels in the lettuce samples collected during all four seasons.

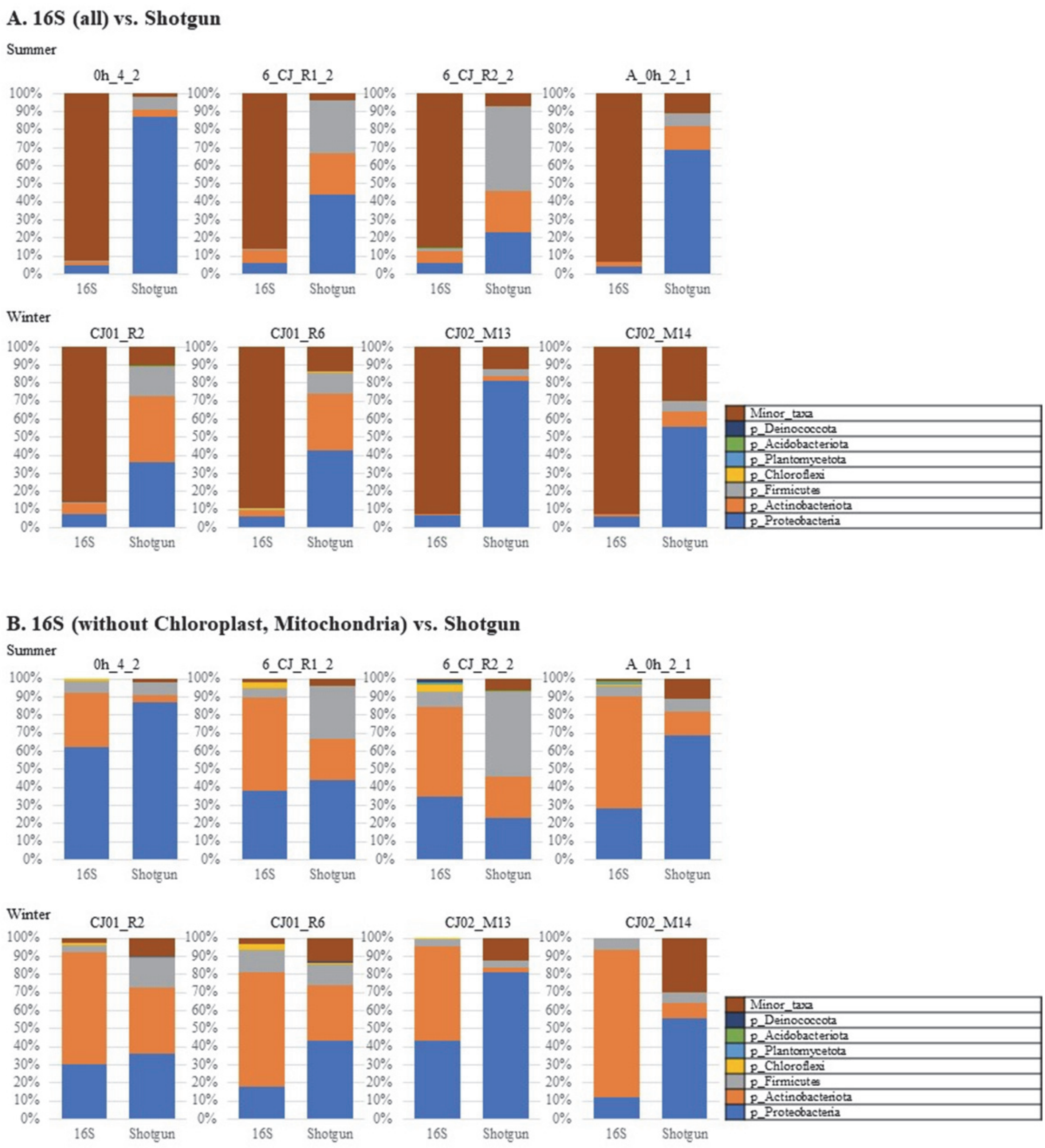

Fig. S2. Comparison of bacterial taxa plots for the same sample. (A) 16S rRNA gene-based sequencing (non-filtered) vs. metagenome shotgun sequencing (B) 16S rRNA gene-based sequencing (without chloroplast-related reads) vs. metagenome shotgun sequencing

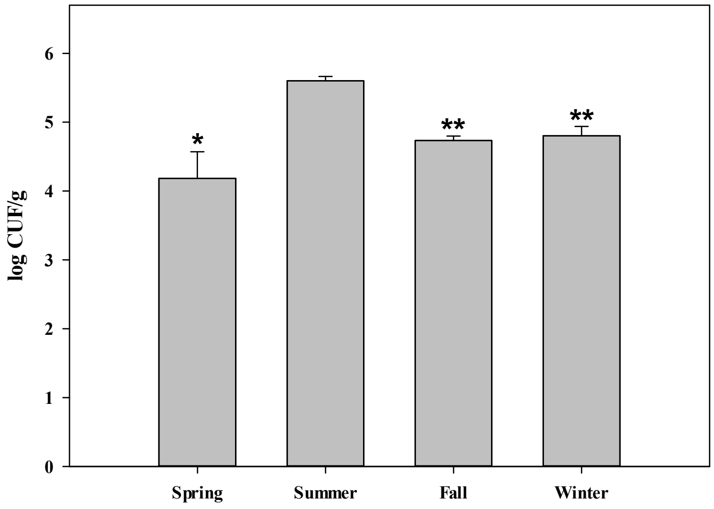

Fig. S3. Cell counts of total aerobic bacteria in lettuce at four seasons. The lettuce samples are as follows: Error bars represent the standard error among the three replicates. *, < p < 0.05; **, p < 0.01 compared with the lettuce of summer group using the Mann-Whitney U test.

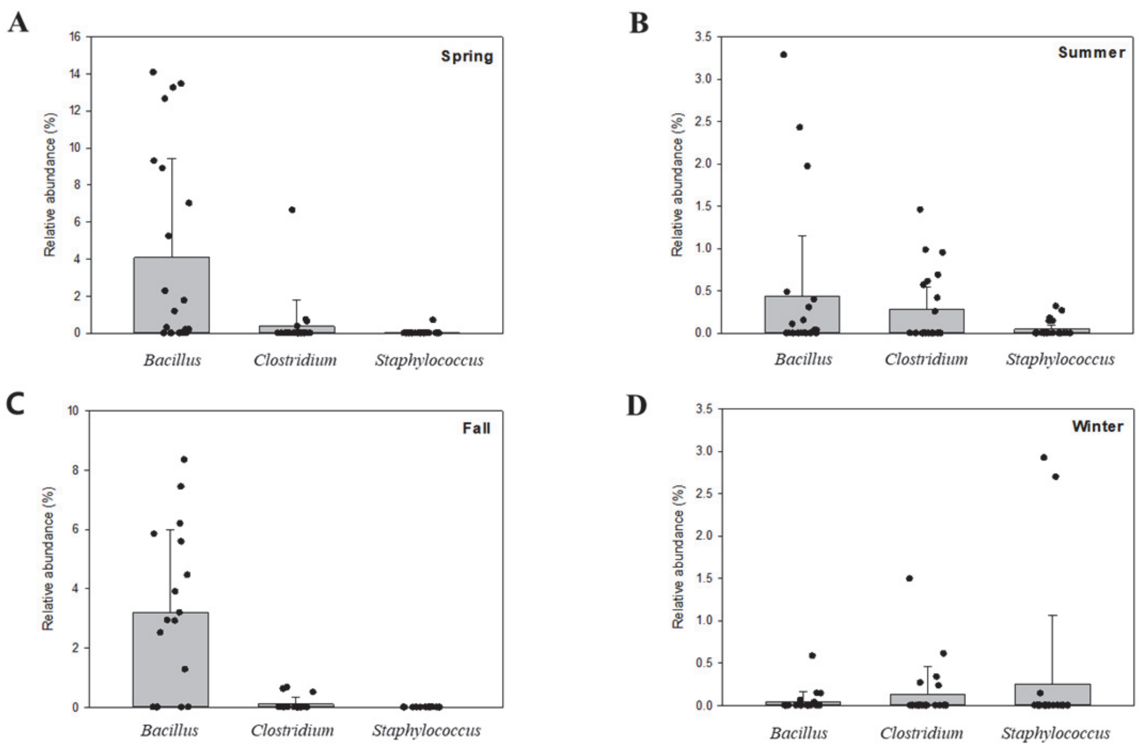

Fig. S4. Bacterial monitoring for potential pathogens in the lettuce samples harvested during different seasons. Three genera of potential foodborne pathogens (*Bacillus*, *Clostridium*, and *Staphylococcus*) were monitored in lettuce samples in **A** spring, **B** summer, **C** fall, and **D** winter.

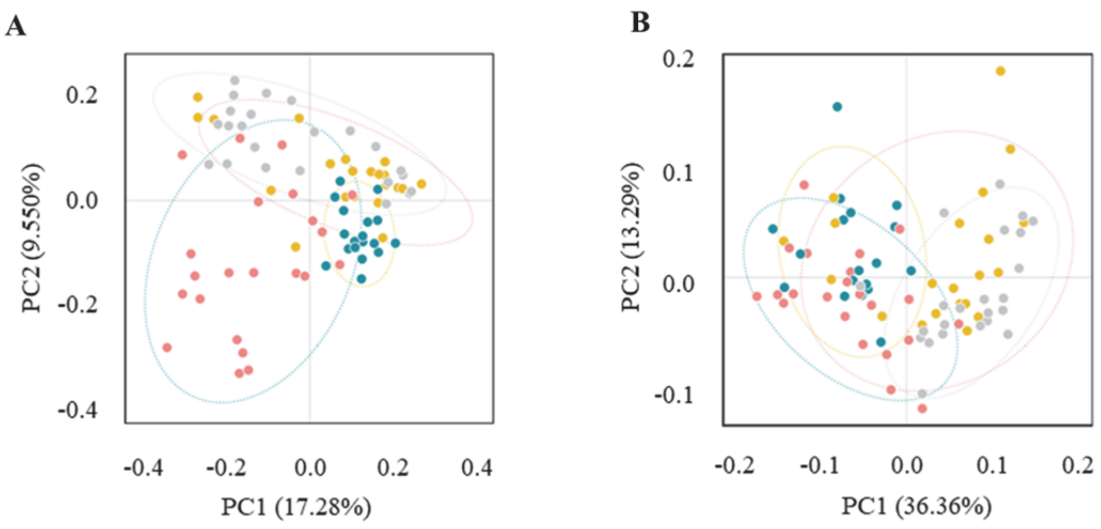

Fig. S5. Comparison of microbial beta diversity in the lettuce samples collected from different seasons. Lettuce microbiota composition across four seasons was compared using a PCoA plot based on the unweighted **A** and weighted **B** UniFrac distance.
